# Supplementary material for: Site-Search Process for Synaptic Protein-DNA Complexes
Source: Int J Mol Sci. 2021 Dec 25;23(1):212. doi: 10.3390/ijms23010212 (PMC8745619; doi:10.3390/ijms23010212)
Supplement: Supplementary file 1 [file ijms-23-00212-s001.zip › ijms-1486646-supplementary.pdf]

Supporting information

Site-search process for synaptic protein-DNA complexes

Sridhar Vemulapalli[1], Mohtadin Hashemi[1], and Yuri L. Lyubchenko[1*]

[1]Department of Pharmaceutical Sciences, College of Pharmacy, University of Nebraska Medical Center, Omaha, Nebraska 68198-6025, United States

[*]Correspondence: ylyubchenko@unmc.edu.

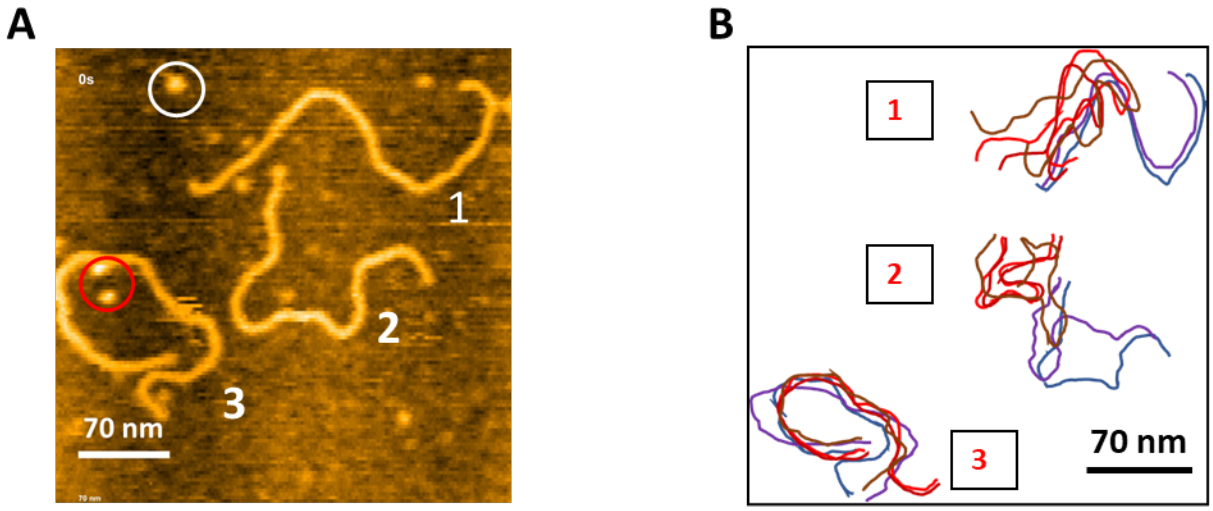

Figure S1. **DNA and SfiI diffusion on the APS-mica surface**. (A) initial frame of Movie S7. SfiI monomer and dimer are indicated by red and white circles, respectively. DNA molecules are numbered 1 through 3. (B) Diffusion of DNA from A on APS-mica. The contour length of the individual DNA strands was traced every 50 frames, each time point is represented in different color.

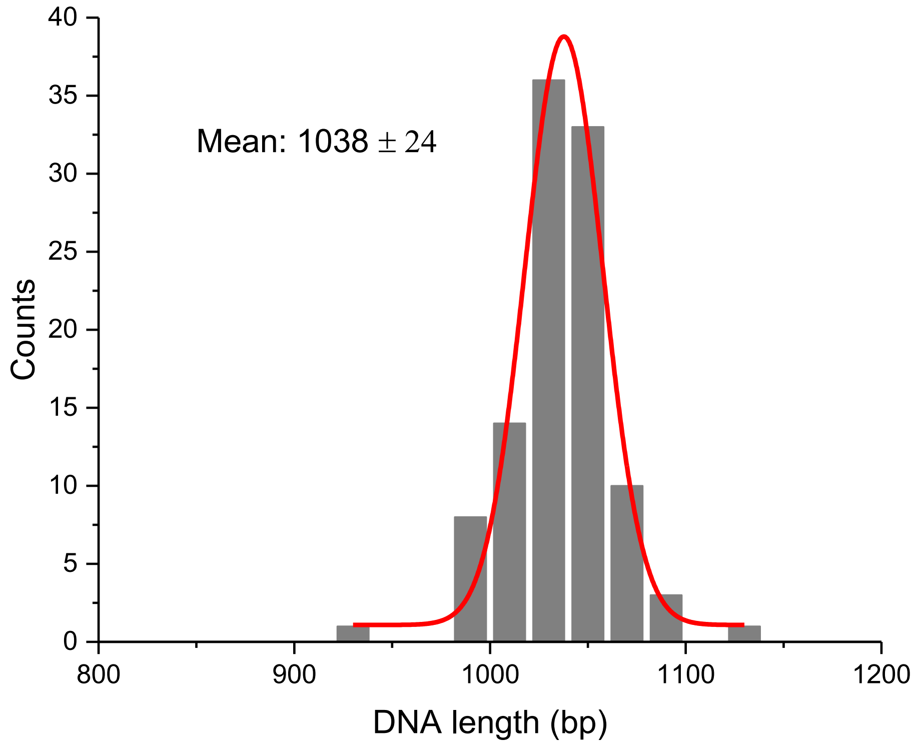

Figure S2. **Statistical analysis of DNA contour length from HS-AFM movie frames (N = 100)**. A single Gaussian was fit to the histogram using bin size of 20 bp, yielding a peak at 1038 ± 24 bp (SD). The DNA length to bp conversion factor was obtained by dividing the mean length in nanometers with the total length of DNA (1036 bp).

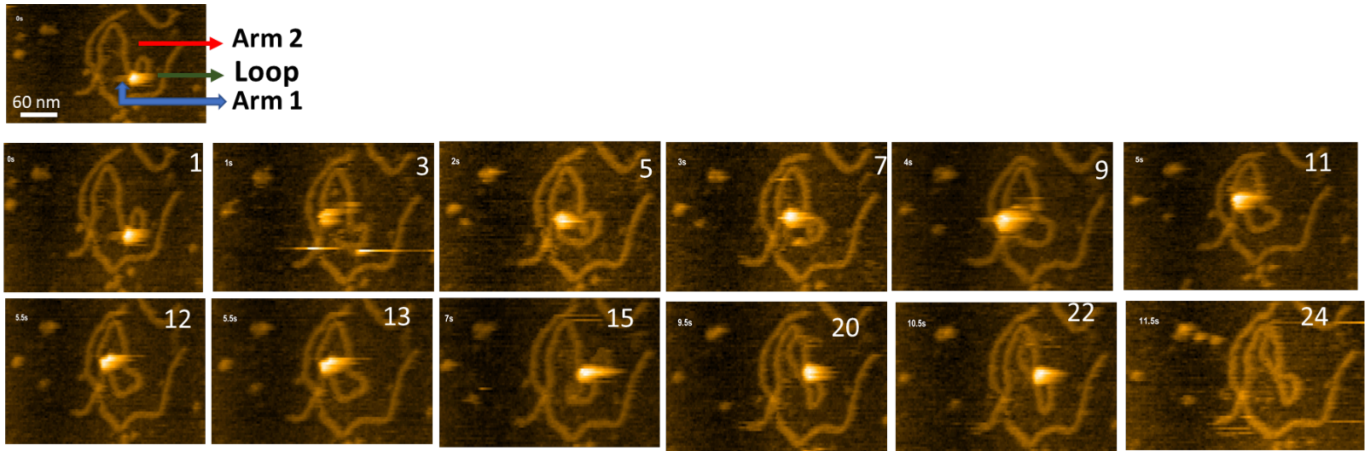

Figure S3. **Site-bound segment transfer pathways of SfiI**. (A) Initial frame of Movie S4. SfiI tetramer can be seen as a bright feature at the center of the loop structure. The loop structure is indicated with a green arrow, the small DNA arm (Arm 1) is shown by a red arrow, and the long DNA arm (Arm 2) is labeled with a blue arrow. (B) Original frames of the HS-AFM movie with important events show the dissociation of the synaptic complex and the formation of a transient loop.

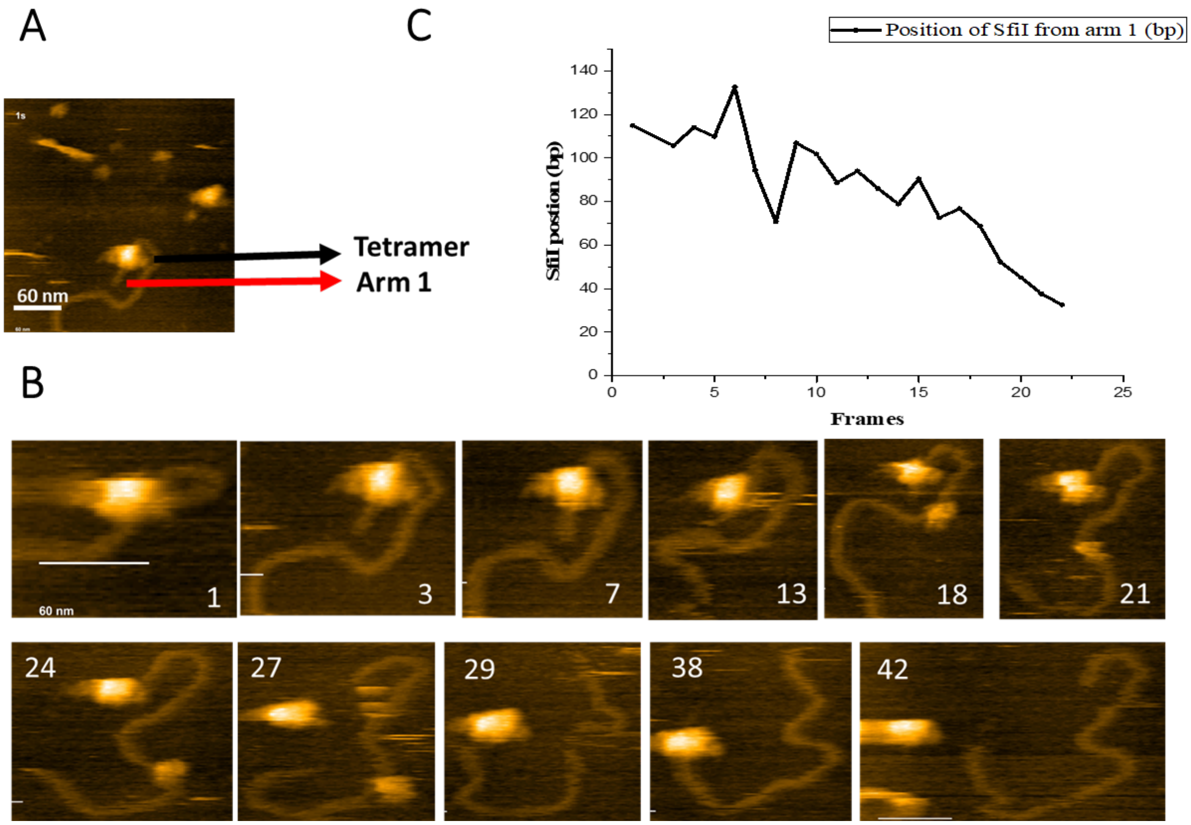

Figure S4. **Sliding of SfiI on DNA**. (A) Initial frame of Movie S6. SfiI tetramer can be seen as a bright feature indicated by the black arrow. The small DNA arm (Arm 1) is labeled with a red arrow. (B) HS-AFM movie frames with important events. The events show the dissociation of the transient loop complex and sliding of the SfiI tetramer along the length of Arm 1, the dissociation of tetramer from the DNA, and SfiI probing the other end of the DNA before finally dissociating from DNA. (C) The graph shows the change in position of the SfiI tetramer with respect to the end Arm 1.

Multimedia

Title and Legend

Movie S1. Time-lapse HS-AFM of synaptic complexes of different loop sizes

Movie S1a. Time-lapse HS-AFM of synaptic complexes of loop size of 254 bp. The size of each frame is 300nm x 300nm. Movie S2a shows the 254 bp loop complex, the dissocaiton of the loop complex, translocation of SfiI, and immediate dissocation into monomers.

Movie S1b. Time-lapse HS-AFM of synaptic complexes of loop size of 532 bp. The size of each frame is 300nm x 300nm . Movie shows the 532 bp loop complex and eventual dissocation of SfiI

Movie S1c. Time-lapse HS-AFM of synaptic complexes of loop size of 786 bp. The size of each frame is 300nm x 300nm . Movie shows the 786 bp loop complex and eventual dissocation of SfiI from the complex as dimers

Movie S2. Time-lapse HS-AFM of SfiI threading pathway

Time-lapse HS-AFM of SfiI threading pathway. The size of each frame is 300nm x 300nm.The movie shows the a synaptic complex of 273 bp. The short DNA arm close to the complex remains unchanged during observation. An increase in the loop size with a consistent decrease in the length of the long DNA arm is seen.

Movie S3. Time-lapse HS-AFM of SfiI threading pathway

Time-lapse HS-AFM of SfiI threading pathway. The size of each frame is 300nm x 300nm.The movie shows a looped complex of ~450 bp. The short DNA arm, closer to the loop stays the same size. A decrease in the loop size with a correlated increase in the length of the long DNA

arm is seen. The tetramer to the left, on the long DNA arm, changes postion at the end of the movie and dissociates from the DNA.

Movie S4. Time-lapse HS-AFM of site-bound segment transfer

Movie S4. Time-lapse HS-AFM of site-bound segment transfer. The size of each frame is 300nm x 300nm. Movie shows SfiI tetramer as a bright feature at the center of the loop structure. Dissociation of the synaptic complex, formation of the transient loop, and return to the initial loop are observed.

Movie S5. Time-lapse HS-AFM of sliding and jumping of SfiI on DNA

Time-lapse HS-AFM of sliding and jumping of SfiI on DNA. The size of each frame is 300nm x 300nm. The movie shows the SfiI-DNA loop complex of ~312 bp. No change in the small arm of the DNA was observed. Loop dissociates and SfiI uses sliding and jumping pathways to translocate on the DNA.

Movie S6. Time-lapse HS-AFM of SfiI sliding on DNA arm

Time-lapse HS-AFM of SfiI sliding on DNA arm. The size of each frame is 300nm x 300nm. The movie shows dissociation of a transient loop complex and sliding of SfiI along the length of small arm of the DNA. SfiI then dissociates from the DNA, probes the other end of the DNA, and eventually dissociates from the DNA completely.

Movie S7. Time-lapse HS-AFM of diffusion of DNA and SfiI on APS-mica surface

Time-lapse HS-AFM of diffusion of DNA and SfiI on APS-mica surface. The size of each frame is 300nm x 300nm. The movie shows the movement of DNA and SfiI across the surface.
